# An Electron Beam Profile Instrument Based on FBGs

**DOI:** 10.3390/s140915786

**Published:** 2014-08-25

**Authors:** Dan Sporea, Andrei Stăncălie, Nicu Becherescu, Martin Becker, Manfred Rothhardt

**Affiliations:** 1 National Institute Laser, Plasma and Radiation Physics, 409 Atomiştilor St., Măgurele, RO-077125, Romania; E-Mail: andrei.stancalie@inflpr.ro; 2 Apel Laser, 15 Vintila Mihăilescu St., Bucharest, RO-060394, Romania; E-Mail: nicu.becherescu@apellaser.ro; 3 Leibniz Institute of Photonic Technology, Albert Einstein Str. 9, Jena 07745, Germany; E-Mails: Martin.Becker@ipht-jena.de (M.B.); Manfred.Rothhardt@ipht-jena.de (M.R.)

**Keywords:** fiber Bragg grating, electron beam, on-line beam profilometry, radiation-hardened optical fiber

## Abstract

Along with the dose rate and the total irradiation dose measurements, the knowledge of the beam localization and the beam profile/energy distribution in the beam are parameters of interest for charged particle accelerator installations when they are used in scientific investigations, industrial applications or medical treatments. The transverse profile of the beam, its position, its centroid location, and its focus or flatness depend on the instrument operating conditions or on the beam exit setup*. Proof-of-concept* of a new type of charged particle beam diagnostics based on fiber Bragg gratings (FBGs) was demonstrated. Its operating principle relies on the measurement of the peak wavelength changes for an array of FBG sensors as function of the temperature following the exposure to an electron beam. Periodically, the sensor irradiation is stopped and the FBG are force cooled to a reference temperature with which the temperature influencing each sensor during beam exposure is compared. Commercially available FBGs, and FBGs written in radiation resistant optical fibers, were tested under electron beam irradiation in order to study their possible use in this application.

## 1. Introduction

Along with the dose rate and the total irradiation dose measurements, the knowledge of the beam localization and the beam profile/energy distribution in the beam are parameters of interest for charged particle accelerator installations when they are used in scientific investigations, industrial applications or medical treatments. The transverse profile of the beam, its position, its centroid location and its focus or flatness depend on the instrument operating conditions or on the beam exit setup.

Various methods have been proposed to evaluate the beam transverse profile: arrays of Faraday cups [1,2]; ionization chamber and imaging plates [3]; micro strip metal detector [4]; pepper-pot device, slit-grid method [5] or rotating slits [6]; Optical Transition Radiation (OTR), Secondary Emission Monitors (SEM) [7,8]; phosphor afterglow [9]; scintillating screen [8,10] or scintillating gas detector [11] and charge coupled device (CCD) camera; flat panel detectors [12], arrays of p-i-n diodes [13] or IonCCD [14]; moving wire scanners [15,16] or vibrating wire scanners (VWS) [17]; Compton-scattered laser light [18]; mesh of scintillating optical fibers [19]; detection of Bremsstrahlung radiation scattered by special foils [20].

The present paper reports the *proof-of-concept* (the design and the implementation) of a beam profile instrument for electron beams, based on an array of fiber Bragg gratings (FBGs). The behavior of commercially available FBGs and FBGs developed in radiation-hardened optical fibers was investigated for the first time under electron beam irradiation, for possible inclusion in the instrument for electron beam profilometry. Tests were carried out under electron beam irradiation at the National Institute for Laser, Plasma and Radiation Physics.

## 2. Operating Principle

In the case of FBGs one or several gratings are inscribed in the optical fiber core by various means able to modulate the fiber-refracting index. As it has a broad spectral band, an optical signal propagates along the optical fiber, a narrow, specific wavelength is reflected, and all other wavelengths are transmitted. This way, the grating acts as a very selective wavelength filter. If a FBG is subjected to external factors (temperature, strain), the period of the grating changes, which implies a modification of the reflected wavelength. The shift of this peak wavelength can be linked to the magnitude of the external stimulus [21]. Under these conditions, the grating operates as an intrinsic optical fiber sensor. The two mentioned effects induce a change in the peak wavelength simultaneously, so, in order to monitor only one of the external parameters, the two effects have to be decoupled.

Exposed to radiation, optical fiber gratings undergo a significant or less significant change in their parameters, depending on the type of the optical fiber (photosensitive optical fibers with various dopants or hydrogen-loaded [22]), the technology used to produce the grating (Type I, Type II, Type IIA FBGs [22], long-period gratings [23,24]) or the characteristics of the irradiation field (gamma-ray [22], neutron [25], proton beams [26]). When such gratings are irradiated under high total irradiation doses they show remarkable radiation resistance [27] or demonstrate some vulnerability to ionizing radiation [28], recommending them for radiation dosimetry.

Based on FBGs' response to temperature under irradiation we designed an instrument for the evaluation of electron beam transverse profile in real time. The paper describes the construction and operation of the developed beam profile instrument. According to our knowledge, this is the first report on FBGs exposed to electron beam irradiation.

## 3. Implementation and Results

**Figure 1.**
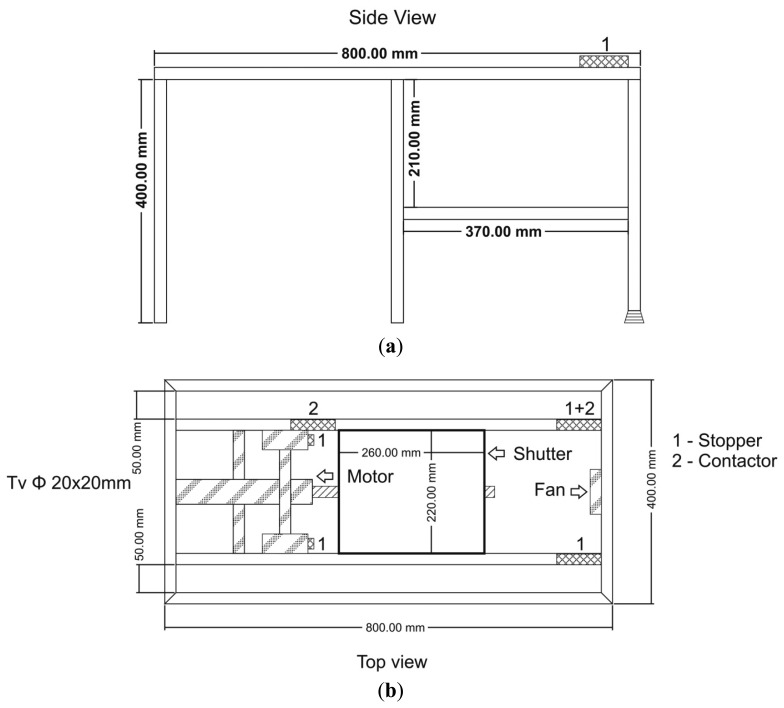

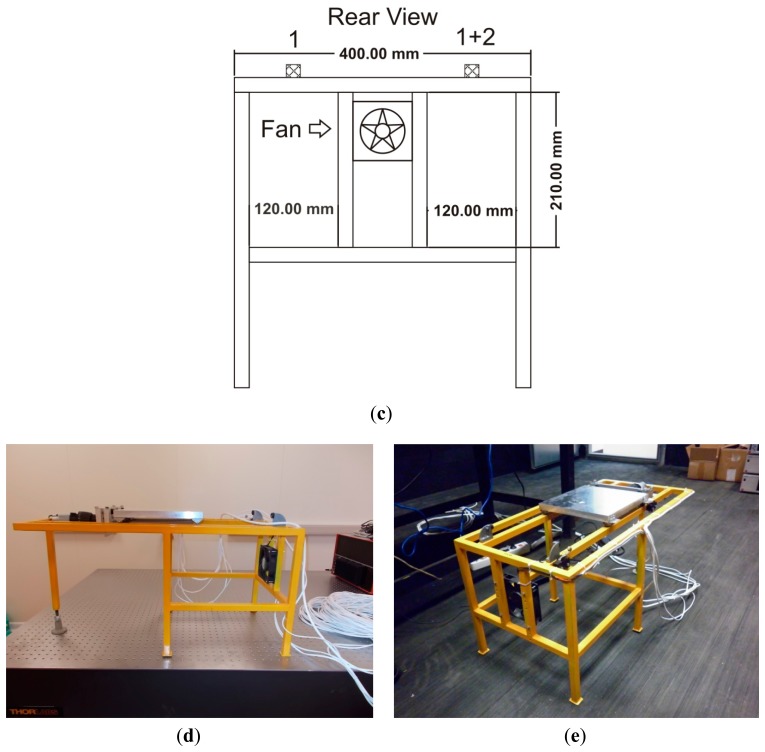
(**a**–**c**) The schematic drawing of the electron beam profile instrument based on FBGs: 1—mechanical stops; 2—limiting switches. (**d**, **e**) the practical implementation of the instrument.

The basic idea of the instrument relies on the wavelength change of FBGs exposed to an electron beam as their temperature increases because of the energy they receive from the incident charged particles. Statistically, spatial mapping of the temperature change is associated with the spatial distribution of the beam energy. In this way, two-dimensional (2D) temperature mapping can be used to evaluate the transversal characteristics of the beam (shape, uniformity, flatness, localization, hot spots detection, etc.). Figure 1 presents a schematic drawing and the practical implementation of the equipment as it was designed to operate, for vertical irradiation geometry in conjunction with the linear accelerator (LINAC) machine developed at the National Institute for Laser, Plasma and Radiation Physics.

From a mechanical point of view, the profile meter has two distinct horizontal planes: in the upper one, a mechanical shutter can be moved along the longitudinal axis of the structure, while the lower plane constitutes the detection plane. In Figure 1c,d the shutter is in the open position enabling the electron beam, which is perpendicular to the two planes from above, to reach the detection plane where the FBGs sensor array is located. The shutter includes a 20 mm thick Al board designed to stop the electron beam from traveling towards the detection plane when closed.

The 2D temperature sensor is located in the detection plane. It includes an array of FBGs embedded into a polystyrene backplane in order to thermally isolate the individual sensors from one another and to prevent any strain from being applied to the gratings during irradiation. The sensors are placed into an array format and are read sequentially by a four-channel sm125 Micron Optics interrogator. The sensor array is exposed periodically to the electron beam, until its temperature reaches a limiting value established by the operator. The upper limit of the temperature depends on sensor characteristics (maximum operating temperature, the dynamic range of the wavelength change with temperature, *etc.*). The limiting temperature value is detected either by monitoring the maximum wavelength shift of all the gratings or with a thermocouple which is simultaneously exposed to the charged particle beam. The mechanical shutter blocks the electron beam incident on the sensor array as the limiting temperature is reached. This way, no more energy is transferred towards the gratings, which are subjected to an accelerated cooling process, as a fan blows air over them. The operator has the possibility to set the lower limit of the sensor temperature, either as a wavelength change or as indicated by the thermocouple. After cooling is achieved, the shutter opens and the exposure of the sensing array to the electron beam energy begins.

The instrument is controlled by a laptop computer through a specially designed interface and a VI developed in LabVIEW. The limits of how far the shutter opens or closes are detected by the controller based on the signals received from two limiting switches. The extreme positions of the shutter are signaled to the operator on the graphical user interface by two virtual light-emitting diodes (LEDs). The cycle can run continuously or can be controlled manually by the operator. During the period the shutter is off (open) the sensors are scanned continuously and data regarding their wavelength values are saved on the laptop hard drive (HD). The acquired data are processed to build three-dimensional (3D) arrays containing information on the sensors' position in the detecting plane and the instant value of their peak wavelength. This way, between subsequent “shutter off” and “shutter on” commands, the spatial change of the beam energy in the transverse plane is recorded. At the end of the data acquisition cycle, the controller displays the distribution of the beam energy, as detected by the gratings array. This map indicates the beam energy at every sensor location by measuring at each point the excursion of the peak wavelength between the lower limit reached by the end of the cooling cycle and the final value read at each location at the end of the exposure process.

The “exposure time” is set by the operator as a function of the beam energy, sensitivity of FBGs and the speed of the heating process (dose rate). The Micron Optics interrogator is controlled through an Ethernet connection, while the temperature from the thermocouple is collected with a NI-cRIO-9211 temperature data logger by a USB connection. The acquisition rate in our case is conditioned by the interrogator scanning frequency (1 Hz), the number of channels (four channels in our application) and the time required for processing information associated with each channel (*i.e.*, the number of gratings per channel). Theoretically, the unit can accommodate up to 80 sensors. A larger number of sensors can be interrogated using an external multiplexing unit (*i.e.*, Micron Optics sm041-408 or sm041-416).

The instrument has no moving parts in the detection plane and, if needed, can operate in vacuum, as the sensors connected in a daisy-chain setup can be coupled with the interrogator through an optical fiber, multi-port vacuum feedthrough device [29]. In such a case, the air cooling has to be replaced by thermoelectric cooling. The instrument can be controlled remotely (in our setup the distance is about 15 m apart from the irradiation zone), so that no other components or devices are exposed to irradiation or to high electromagnetic interferences. Only the FBGs, the thermocouple and the mechanical shutter are placed in the irradiation area.

The spatial resolution, which can be achieved with such a profile meter, is set along one axis by the distance between two sensor rows (located 3–5 mm apart) and along the other axis by the length of individual FBGs (which in our case was either 12 mm for commercial the products we used, or 4 mm for the custom designed components). The dynamic range for the beam energy detection with FBGs depends on the span between the low and high temperature values. In our study, we set the low temperature value to 18 °C, while the highest temperature was 65 °C, as allowed by the upper limit imposed by the FBGs used. An extended range can be achieved between 18 °C and 200 °C if high operating temperature gratings are used instead. This dynamic range ensures a linear response of the sensor over the temperature range. The resolution of the acquired signal is 0.1 °C, corresponding to the best spectral resolution provided by the interrogator. The temporal resolution of the equipment is dependent on the electron beam dose rate. In the experimental setup we used (an increase of the FBGs temperature up to 37 °C and the air flow provided by a PC fan) the cooling time was about 1–2 min. The presence of “hot spots” or non-uniformities of the beam energy distribution are locally responsible for the upper temperature limit. The mechanical shutter “turn-on” and “turn-off” times are in the order of 30 s and can be adjusted by the operator according to the experiment needs.

**Figure 2.**
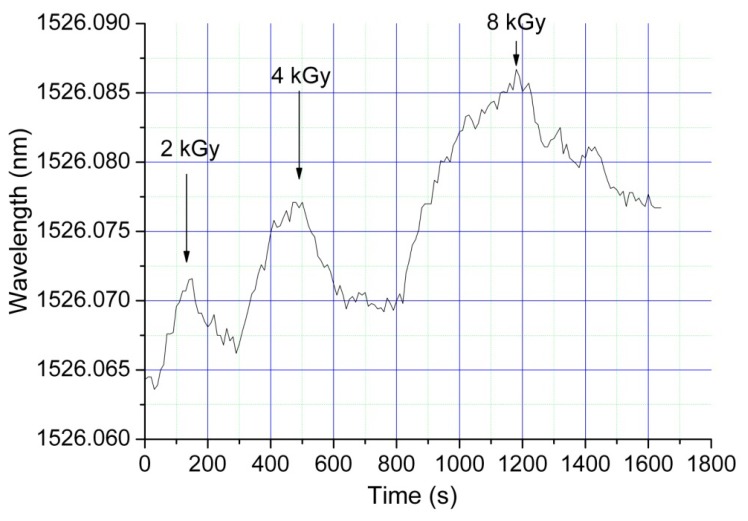
The effect of the bias during the wavelength reading, after subsequent exposure of the same sensor to different doses, in the case of natural cooling by convection.

The reason for the inclusion in the setup of the shutter and the fan is to eliminate the bias present after each exposure, as the sensor array needs quite a long time to cool down based on only convection. Figure 2 illustrates this bias when the sensor array is exposed to increased total doses and the cooling is provided only by air convection. In the illustrating curves, the positive slopes correspond to the FBGs exposure to the electron beam, while the negative slopes reflect the FBGs cooling, by natural convection as the irradiation is stopped. As can be noticed from the acquired data, it is difficult to naturally cool the sensor array after the exposure stops. The presence of this biasing level in the wavelength readout leads to a long time interval between exposures, as requested by the natural convection. By closing the shutter, the irradiation of the sensors is stopped and they are simultaneously cooled to the reference temperature of 18 °C. This approach also has the advantage of checking the beam quality by obstructing the beam for very short durations. The force cooling is also required as the temperature in the operating room varies when the accelerator is subjected to various operating conditions. The shutter can be used in the “on” (closed) position as support for the samples to be irradiated, so the operator can switch between sample irradiation and beam quality check.

For all measurements, we considered only the wavelength shift of each sensor against the initial level when the measurements started. The presence of the bias signal along with the imposed upper temperature during the irradiation limits the dynamic range of the instrument. A higher dynamic range can be achieved by: (i) blocking the electron beam simultaneously by force cooling the sensors; (ii) using FBGs able to operate under higher temperatures.

Whatever the situation is, during the exposure of the sensor array, data are acquired continuously at the rate of one scan per second. With this approach, the full “history” of the electron beam energy distribution changes during the monitoring process is available to the operator, who can correlate these changes with the operating conditions initially set (beam current, magnetron voltage, voltage on the injection section of the machine, *etc.*).

In order to use FBGs as sensors in the proposed electron beam profile instrument and to assess their degradation under this type of radiation exposure we tested some commercially available FBGs and a FBG from a set written in a radiation-hardened optical fiber. Gratings in single mode (SM) andnon-photosensitive radiation-hardened fibers were written by a two-beam interferometer and deep ultraviolet femtosecond laser radiation [30]. The produced FBGs have a peak wavelength between 1545 nm and 1554 nm and a reflectivity varying between 30% and 90%. The commercially available products are from Technica SA, written in SMF-28 optical fiber, and operating at λ = 1516 nm, 1526 nm, 1536 nm, 1546 nm, each grating having a length of 12 mm, and the following parameters: reflectivity > 80%; BW@3 dB < 0.3 nm; Side Lobe Suppress Ratio (SLSR) > 15 dB; Acrylate recoating.

**Figure 3.**
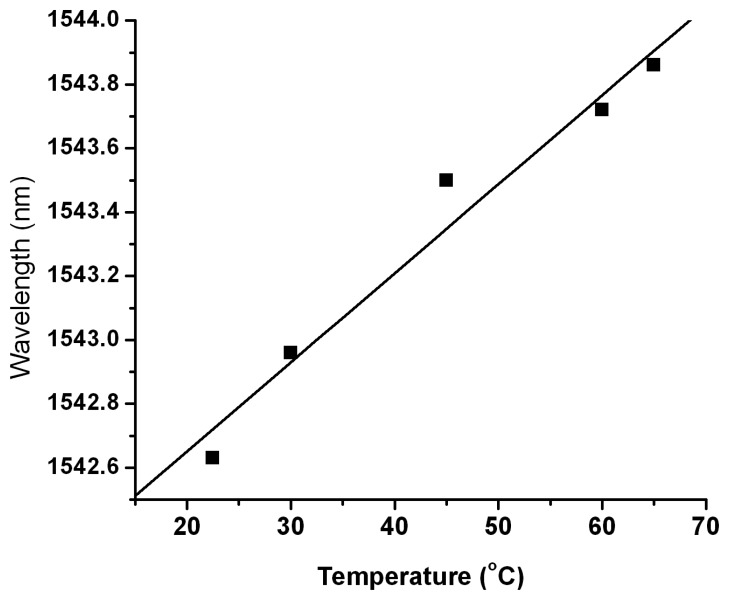
Calibration curve of the fiber Bragg gratings (FBG) sensor.

For the evaluation of the FBGs degradation under electron beam irradiation, tests were done by subjecting the gratings to temperature cycling, heating and cooling them from room temperature to 65 °C, in a temperature-controlled chamber type Memmert UE 400, in order to evaluate their behavior after irradiation. These tests were run initially and between the irradiation steps. During the temperature cycling tests, we monitored the Bragg wavelength change and the full spectrum of the gratings. The chamber temperature was increased linearly via commands from the controller we developed, while the cooling was done through natural convection. For both the heating and the cooling process, the controller acquired data from the NI temperature data logger and from the Micron Optics interrogator. An example of a calibration curve used for the evaluation of the temperature sensitivity for one of the tested FBG is given in Figure 3. After each test, the modifications of the full width at half maximum (FWHM), the temperature sensitivity and the SLSR were estimated. For the total irradiation doses reached during the irradiation campaigns no significant changes of these parameters were noticed.

**Figure 4.**
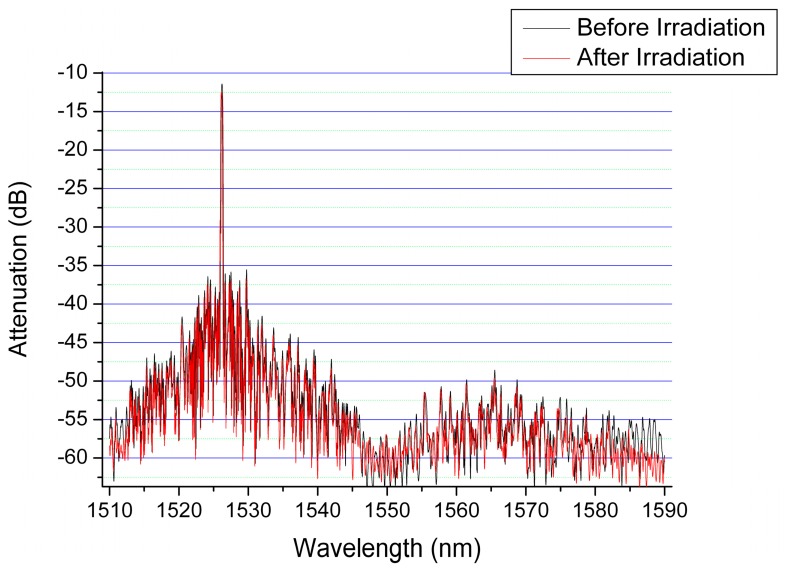
The evaluation of the commercially available FGB parameters: (black) before the irradiation; (red) after exposure to electron beam, measurements done at 23 °C.

Figure 4 compares the spectral characteristics of a commercial available FBG before any irradiation and after it was subjected to a cumulated total dose of 292 kGy, both measurements being done under strict temperature control at 23 °C. As can be noticed, even for extensive exposure to the electron beam for over 130 min, no visible degradation of the FBG parameters was observed. The total dose present in the operation state of the instrument we designed was much lower than the dose received during the reliability tests.

The description of the beam profile instrument operating principle and the requirements of the tests performed between the irradiation steps underline the two sets of functions which have to be performed by the controller: (i) to control and to measure the chamber temperature and to acquire the spectrum of a single FBG during the heating-cooling processes; (ii) to collect information on the Bragg wavelength shift from the sensors included in the detection array; to monitor the temperature in the detection plane during the exposure and the cooling of the sensor array; to switch the shutter and the cooling fan “on” and “off” as appropriate; to save the acquired data on HD; to display the recorded data as a 2D/3D representations; to make possible the recall of the stored data.

Based on these needs, the controller assists the operator with both running the instrument as an electron beam profile meter and with testing the possible irradiation-induced degradation of the gratings. To perform these tasks, the controller includes two main units: (i) a custom developed board, a high-side switch 2n06lh5 with the gate driver, controlled by 5 V; a dual full bridge driver able to provide 4 A current to operate a DC motor driving the shutter; the freewheeling diodes for motor overload protection; the 5 V regulator used to monitor the proximity switches states, switches which detect the limits of the shutter travel; (ii) an Arduino UNO board (ATmega328 microcontroller; 32 KB flash memory and 1 KB EEPROM).

The Arduido board supervises the operation of the controller and of the motor, which drives the shutter (sense, speed, detection of the limits). The control of the motor by the Arduino UNO board is done using the LIFA program (LabVIEW Interface for Arduino).

Four graphical windows are available to the operator: “Shutter”; “Thermocouple”; “Oven”; “Interrogator Data Acquisition”. Seven buttons on the main window enable the operator to enter commands: (a) “Start cool down”—overwrites any previous command and starts the air flow over the sensors matrix; (b) “Stop cool down”—overwrites any previous command, moves the shutter into the off position and stops the cooler; (c) “STOP Shutter”—stops the shutter at the current location; (d) “Start/Reset Motor Control & Temperature Measurement”—initiates all instrument functions (temperature recording over time, the cooling-exposure cycle, recording of detected peak values, *etc.*), and does not affect final displayed data; (e) “Start Acquisition”—initiates the display of the acquired data, in graphical form; (f) “Stop Acquisition”—stops data acquisition from the interrogator; (g) “STOP”—stops the program running and save all data under a name selected by the operator.

At the end of the exposure period the beam profile instrument displays on the screen the final results corresponding to the energy (associated with the total dose) received at each sensing location during the data acquisition process. These data represent only the change of the temperature recorded by each FBG sensor (as a wavelength shift), measured between the low temperature limit and the final temperature. At this moment, the operator has the choice either to allow the software to continue running the irradiation-cooling cycles or to interrupt the process and display additional frames from those acquired during the irradiation. As the frames are time series coded, the operator has the possibility to visualize the evolution of the beam energy transverse distribution at different moments in time.

**Figure 5.**
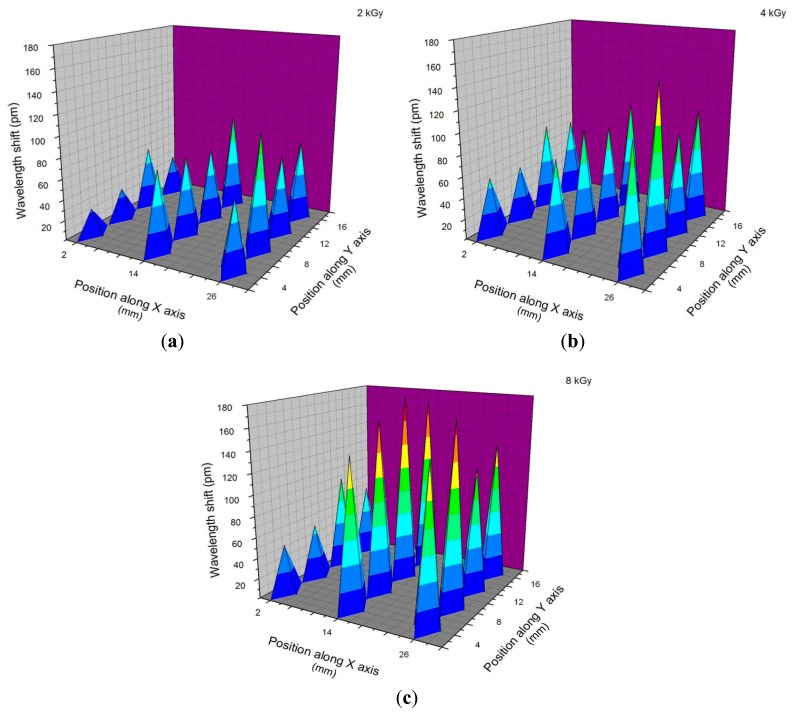
Illustration of the three dimensional (3D) reconstruction of the deployed energy on the FBG array at: (**a**) 2 kGy; (**b**) 4 kGy; (**c**) 8 kGy.

Figure 5 illustrates the reconstructed results during the tests, in the case of commercially available FBGs arranged in four rows at a distance of 4 mm from one another. The temperature was monitored with the thermocouple placed in the same position, and various total doses were used to expose the sensor array. By using this detection geometry, a mapping of a large field can be reconstructed. In our present implementation, up to 120 frames are stored and can be recalled. The number of frames depends on the dose rate and exposure time. Data presented in Figure 5 corresponds to the final wavelength shift values reached after exposure to 2 kGy, 4 kGy and 8 kGy. From the acquired data, a 3D animation of the beam transverse energy profile can be generated, illustrating the spatio-temporal variation of the signal generated by the sensor array, as it is subjected to subsequent total irradiation doses. The supplementary file “Animation_3frames.tif” is an example of the electron beam profile temporal change reconstructed for the three doses. For visualization the file has to be open with Internet Explorer.

To evaluate the resolution of the instrument, we have to mention that for the tested irradiation geometry, the maximum wavelength change achieved (180 pm) is associated with 8 kGy. Considering the wavelength accuracy of the interrogator to be 1 pm, for our setup the best detection resolution is about 44 Gy. As proved by the calibration curve in Figure 3, the instrument response is liner up to 65 °C for the FBGs we used. In the presented example, the temperature increases up to 28 °C, which is in the linear range of operation of the instrument.

The irradiation tests were run at the Linear Accelerator facility of the National Institute for Lasers, Plasma and Radiation Physics. The electron accelerator, of the traveling wave type, has the following characteristics: pulsed operation; macropulse length—4 μs; mean electron energy—6 MeV; electron beam current—1 μA, pulse repetition rate—100 Hz; pulse duration—3.5 μs, dose rate—2 kGy/min. The beam diameter in the irradiation plane was about 100 mm × 100 mm.

**Figure 6.**
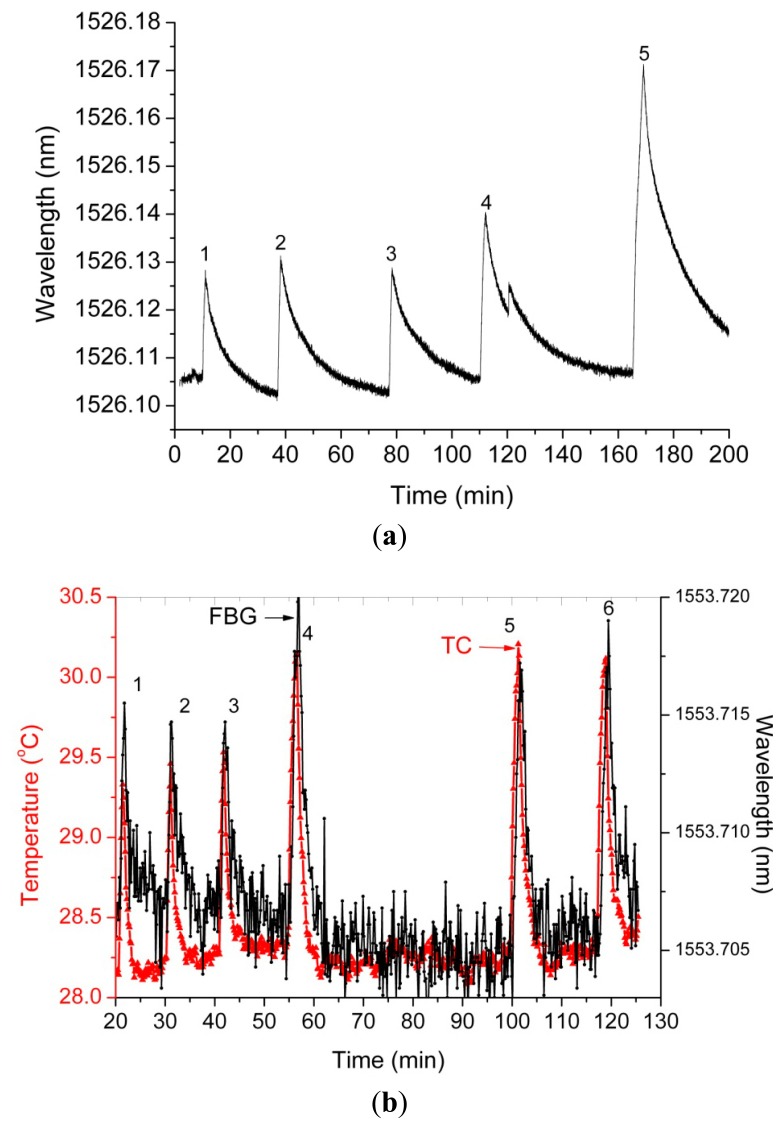
Results of the exposure to the electron beam of: (**a**) a commercially available FBG; (**b**) a FBG (black curve) inscribed into a radiation-hardened optical fiber (RAD HARD FBG). The temperature in the detection plane was monitored by a thermocouple (TC, red curve).

Examples of the results of the irradiation tests carried out on a commercially available FBG and on one FBG inscribed into a radiation-hardened optical fiber are provided in Figure 6. They are used here to derive some conclusions on the radiation resistance of the optical fibers used in the implementation of the electron beam profile instrument. In both cases, the irradiation was done by switching on and off the electron beam.

In the first situation, after the irradiation stops, the cooling of the grating was achieved by natural convection, and no force cooling was employed. For this reason, the wavelength recovery post irradiation is not complete. On the other hand, as the tests were done without any insulation against ambient temperature changes, because the electron beam was propagating in the air, the final temperature reached by the grating was dependent on the ambient temperature variation in the testing facility. This ambient temperature changed as the subsystems composing the LINAC warmed up. In the meantime, the operation of the machine by switching it on and off involved some instabilities related to the transient regime of the accelerator during the start up. For this reason, we adopted a solution with shutter blocking the beam and force cooling. A higher stability and reproducibility of the results can be achieved with this solution. Even operating under natural convection, the detecting system proves a good reproducibility as indicated by the graph in Figure 6a, where points 1, 2 and 3 correspond to a 2.5 kGy total dose, point 4 to 3.5 kGy and point 5 to 6.7 kGy. Nevertheless, according to the proposed implementation, useful information is derived from the difference between the wavelength values at the end and at the beginning of the exposure in every single sensor's location. By their nature, our measurements are relative and not absolute. The proposed method is used for beam profile monitoring, not for radiation dosimetry. This method makes possible the evaluation of the spatial distribution of the beam energy, which can be associated with the total dose involved. The temporal variation of this energy provides information, which can be related to the dose rate.

The thermocouple was used: (i) to monitor the chamber temperature during the calibration process; and (ii) to evaluate the behavior of different types of FBGs during the irradiation tests. Any differences between the FBG and the thermocouple output signals during the exposure to the electron beam signalize a possible degradation of the FGB, as it was irradiated.

The results from Figure 6b indicate a very reproducible recovery of the wavelength inscribed in radiation-hardened optical fibers (RAD HARD) FBG. The wavelength changes strictly follow the temperature variation as indicated by the thermocouple (TC). In this case, points 1, 2, and 3 correspond to a total dose of about 2 kGy, while points 4, 5, and 6 are associated with a total dose of about 4 kGy. The differences between the two cases are explained by the fact that they were not measured during the same irradiation session; however the sensors were placed in the same location. This fact is visible in Figure 5, which clearly indicates the spatio-temporal variation of the incident energy (associated with the dose rate). For the same sensor, the subsequent irradiation and cooling cycles were performed by keeping the sensor in the same position with respect to the incident electron beam. Some differences present in the wavelength shift for approximately the same exposure duration can be explained by the fluctuation of the dose rate of the electron beam. A higher dynamic range is possible if the sensors are cooled down to 15 °C, while the maximum allowed temperature is 65 °C. A higher upper limit of temperature can be associated with longer exposure times, which correspond to higher total doses or dose rates to be monitored.

The problems solved by the proposed equipment are related to the availability of information on the temporal variation of the deposited energy in different locations of the irradiation plane and on the spatial non-uniformity of the energy in the beam cross section. An advantage of our solution is the continuous, multipoint acquisition of data over the beam cross section. By the end of an exposure period, a value proportional to the total energy associated with the total dose received in each sensor location is available (information on beam spatial uniformity is obtained). By recalling intermediary data saved for each location, the operator can assess information associated with the temporal stability of the beam energy.

During the irradiation sessions, the shutter is closed and the samples to be irradiated are placed on the top of the shutter plate. With this setup, when the beam quality has to be checked, the irradiation is stopped for a short time and the beam is sampled by the FBGs array.

## 4. Conclusions and Future Work

The beam diagnostics method and equipment we present here has several advantages over classical approaches: it is a non-invasive method; it can be operated remotely and under high electromagnetic fields; vacuum operation is easily achieved; daisy-chained and wavelength multiplexing setups make the extension of the number of sampling locations possible; and no moving parts are placed in the detection plane. As our tests have proven, no significant damage was noticed in the FBGs exposed to electron beams. In any case, even if such effects are present, the method accommodates some corrections as only relative temperature changes or wavelength variations, respectively, are recorded for data processing. Besides that, periodical recalibrations are done using the temperature tests run in the temperature-controlled chamber. Being based on an intrinsic optical fiber sensor, the instrument requires no alignment. As compared to some classical methods, the newly proposed one is a real time measurement device. It has the advantage that it almost continuously records the energy distribution in the transversal cross section of the charged particle beam. The intermediary energy distributions can be recalled and compared and associated with the changed operating conditions of the accelerator. The setup by its principle is fire- and explosion-safe to operate. The demonstrated production of FBGs in radiation-hardened optical fibers extends the possibility to operate this beam profile instrument under ionizing radiation conditions.

As these investigations and the associated implementation have acted as a proof-of-concept, we will focus our efforts in the near future to refine the equipment design in order to achieve a better spatial resolution (shorter and closely located FGBs inscribed in the same optical fiber) and to further investigate other types of FBGs, which can be embedded into the detecting system. The use of optical fibers designed for high temperature operation can assure an extended lifetime and a higher dynamic range. In addition, more tests on custom designed FBGs inscribed in a standard optical fiber and radiation-hardened optical fibers have to be performed.
